# The Meganuclease I-SceI Containing Nuclear Localization Signal (NLS-I-SceI) Efficiently Mediated Mammalian Germline Transgenesis via Embryo Cytoplasmic Microinjection

**DOI:** 10.1371/journal.pone.0108347

**Published:** 2014-09-24

**Authors:** Yong Wang, Xiao-Yang Zhou, Peng-Ying Xiang, Lu-Lu Wang, Huan Tang, Fei Xie, Liang Li, Hong Wei

**Affiliations:** 1 Department of Laboratory Animal Science, College of Basic Medical Sciences, Third Military Medical University, Chongqing, China; 2 China Three Gorges Museum, Chongqing, China; University of Minnesota Medical School, United States of America

## Abstract

The meganuclease I-SceI has been effectively used to facilitate transgenesis in fish eggs for nearly a decade. I-SceI-mediated transgenesis is simply via embryo cytoplasmic microinjection and only involves plasmid vectors containing I-SceI recognition sequences, therefore regarding the transgenesis process and application of resulted transgenic organisms, I-SceI-mediated transgenesis is of minimal bio-safety concerns. However, currently no transgenic mammals derived from I-SceI-mediated transgenesis have been reported. In this work, we found that the native I-SceI molecule was not capable of facilitating transgenesis in mammalian embryos via cytoplasmic microinjection as it did in fish eggs. In contrast, the I-SceI molecule containing mammalian nuclear localization signal (NLS-I-SceI) was shown to be capable of transferring DNA fragments from cytoplasm into nuclear in porcine embryos, and cytoplasmic microinjection with NLS-I-SceI mRNA and circular I-SceI recognition sequence-containing transgene plasmids resulted in transgene expression in both mouse and porcine embryos. Besides, transfer of the cytoplasmically microinjected mouse and porcine embryos into synchronized recipient females both efficiently resulted in transgenic founders with germline transmission competence. These results provided a novel method to facilitate mammalian transgenesis using I-SceI, and using the NLS-I-SceI molecule, a simple, efficient and species-neutral transgenesis technology based on embryo cytoplasmic microinjection with minimal bio-safety concerns can be established for mammalian species. As far as we know, this is the first report for transgenic mammals derived from I-SceI-mediated transgenesis via embryo cytoplasmic microinjection.

## Introduction

Genetic modification of mammalian genomes is of great importance for bio-medical researches such as deciphering gene functions, investigating disease mechanisms and searching and validating therapeutic targets, and also a potential method to generate farm animals with improved economic traits for agricultural purposes.

Mammalian genetic modification includes transgenesis, gene disruption and random mutation of genomes. Gene disruption was once a sophisticated and labor-intensive process which was based on DNA homologous recombination (HR) in embryonic stem cells (ESCs). However, this DNA HR-based technology achieved very limited success in mammalian species other than mice due to the lack of ESCs derived from these species. Recently, with the development of powerful site-specific engineered endonucleases(EENs), especially Zinc Finger Nucleases(ZFNs) [1]–[4], Transcription Activator-like Effector Nucleases (TALENs)[5]–[10] and Clustered Regularly Interspaced Short Palindromic Repeats/CRISPR-associated system 9 (CRISPR/Cas9) [11]–[15], which are capable of disrupting genes efficiently by making double strand breaks (DSBs) at target sites, gene disruption has become a much more efficient and convenient process which is independent on ESCs and achieved significant success in mammalian species other than mice. Random mutation of mammalian genomes is regularly efficient using powerful chemical mutagens such as ENU or insertional viral vectors. In contrast, mammalian transgenesis, especially for species other than mice, remains to be further optimized.

Transgenesis is a process of adding exogenous and (or) artificially constructed genes to animal genomes, which is indispensable for generating mammalian models with gain of functions for bio-medical researches or genetically modified farm animals with additional economic traits. Currently, the available technologies for mammalian transgenesis include embryo pronuclear microinjection, somatic cell nuclear transfer (SCNT) using transgenic cells as nuclear donors, sperm-mediated gene transfer (SMGT), lentiviral transgenesis using retro-viral vectors derived from lentiviruses as vehicles to deliver transgenes into animal genomes and transposon-mediated gene transfer. Embryo pronuclear microinjection is a reliable and traditional method to produce transgenic mammals, but the inaccessibility to pronuclear of many mammalian species other than mice and the low efficiency of transgene integration largely limits its effectiveness and utility [16], [17]. SCNT is a reproducible method to produce transgenic mammals, but SCNT is a sophisticated and complex procedure with a rather low efficiency [18], [19] and a large number of oocytes are needed. Practically, many mammalian species of biological or biomedical importance, such as non-human primates or other none-economic animals, are not able to be cloned due to the lack of regular ovary sources. Besides, the unpredictable abnormalities related to cloned individuals limit the usage of resulted transgenic animals to model human diseases, and the antibiotic resistant genes, the necessary selection markers for transgenic nuclei donor cell culture which are finally added into the genomes of resulted transgenic individuals by SCNT process, brings additional uncertainties for the application of derived transgenic animals. SMGT is reported to be a simple and inexpensive method for transgenic animal production, however extremely variant data has been reported from different labs and the highly unstable outcome of this technology limits its application. Lentiviral transgenesis has been recognized as an extremely efficient method to generate transgenic animals of different species [20]–[22]. However, the preparation of high titre lentiviral particle suspensions is a complicated procedure and the viral vectors integrated into animal genomes are of bio-safety concerns. Transposon systems have been used for animal transgenesis [23]–[27], but transposons are mobile genetic elements, and the derived transgenic animals are of similar bio-safety concerns as those derived from lentiviral transgenesis.

On the basis of these points mentioned above, it is valuable to develop an efficient, simple, and species-neutral transgenesis technology for mammals, which is of minimal bio-safety concerns being without the involvement of viral or mobile vectors and independent on SCNT process or the accessibility to embryo pronuclear. The meganuclease I-SceI, which is derived from the mitochondria of *Saccharomyces cerevisiae* and has a long (>18 bp) recognition sequence that does not exist in animal genomes naturally, has been effectively used to facilitate trangenesis in fishes via embryo cytoplasmic microinjection [28]–[32]. However, in this study we found that the native I-SceI molecule failed to efficiently facilitate transgenesis in mammalian embryos as it did in fish eggs after cytoplasmic microinjection along with the plasmids of transgene vector containing two inversely flanking I-SceI recognition sequences, suggesting that in mammalian embryos, the native I-SceI molecule did not exhibit the efficacy on transgenesis in the same way as that in fish eggs. By adding a mammalian nuclear localization (NLS) signal to the N-terminus of I-SceI molecule, the I-SceI molecule containing NLS (NLS-I-SceI) was found to be capable of translocating DNA fragments from mammalian embryo cytoplasm into nuclear, and the I-SceI recognition sequence-containing transgene vector plasmids, which was injected into cytoplasm along with NLS-I-SceI mRNA, exhibited expression in both mouse and porcine embryos. By transferring the embryos cytoplasmically co-injected with NLS-I-SceI mRNA and the transgene plasmids into synchronized female recipients, transgenic founder animals were efficiently generated and transgenes were found to be capable of germline transmission. These data suggested that using the NLS-I-SceI molecule, a simple, efficient and species-neutral transgenesis technology, which was based on embryo cytoplasmic microinjection and without the involvement of viral or transposon vectors, can be established for mammals.

## Materials and Methods

### Animals

Mice of FVBN inbred strain and Bama minipigs, which are of one local minipig strain in China, were used in this study. The mice were purchased from SLAC Laboratory Animal Co., Ltd (Shanghai, China) and maintained under specific pathogen-free conditions in Laboratory Animal Centre of our university. The minipigs used in this study were derived from the closed colony regularly maintained in Laboratory Animal Centre. All the protocols involving the use of animals were approved by the Institutional Animal Care and Use Committee of Third Military Medical University (Approval ID: SYXK-PLA-2007036).

### Construction of NLS-I-SceI molecule and transgene vector

The NLS-I-SceI molecule was constructed by adding a modified version of 3×SV40 NLS sequence containing a HA epitope to the N-terminal of the native I-SceI molecule. The coding sequence for NLS-I-SceI, of which the initiation codon was surrounded by a kozak sequence for optimal translation initiation and the codons were optimized for both pigs and mice, was artificially synthesized and subcloned into the mammalian expression vector PCI (Promega) downstream T7 promoter, and the resulted vector was designated as PCI-T7-NLS-I-SceI in this article. For the convenience of transgene vector construction, an intermediate vector designated as p2IS was constructed by subcloning a synthesized DNA fragment containing a long multi cloning sites (MCS) inversely flanked by two I-SceI recognition sequences into pUC18 vector at the two restriction sites BsmBI and SapI to substitute the original MCS region. To construct the transgene vector used in this study, a DNA fragment containing human Ubiqutine C (UBC) promoter, eGFP CDS and a poly (A) signal sequence was cut off from FUGW plasmid (Addgene, #14883) using the two endonucleases PacI and PmeI and then subcloned into p2IS vector at the same two restriction sites, and the resulted transgene vector was designated as p2IS-UBC-eGFP.

### Preparation of mRNA

NLS-I-SceI mRNA was prepared by *in vitro* transcription using linearized PCI-T7-NLS-I-SceI plasmid as templates. The plasmid was linearized by restrictive digestion at the ClaI site which was located downstream NLS-I-SceI CDS. After complete digestion, the reaction system were treated with proteinase K (100 µg/mL) and SDS (0.5% (v/v)), and then further treated with one equal volume of phenol:chloroform mixture. After centrifuge at 12000 g, 4°C for 10 min, the supernatant was carefully collected and the DNA was precipitated by adding 2.5 volumes of ice-cold absolute alcohol and one tenth volume of RNase-free 5 M NaAc solution. After washing in 75% alcohol, the DNA precipitate was finally dissolved into RNase-free deionized water after drying. Using the purified linearized plasmids as templates, NLS-I-SceI mRNA was produced by *in vitro* transcription using the mMESSAGE mMACHINE@T7 Ultra Kit (Life Technologies, AM1345) as described in the manual. After transcription was terminated, 1 µL of transcription products was saved prior to poly(A) tailing as a control to assess the tailing quality after poly(A) tailing procedure was completed. To prepare purified mRNA for embryo microinjection, the poly(A)-tailed mRNA products were recovered from reaction system using RNeasy Mini Kit (Qiagen, 74104) and eluted with RNase-free deionized water. The quality of mRNA samples was assessed by agarose gel electrophoresis.

### Embryo microinjection, observation and transfer

The circular or linearized transgene vector plasmids p2IS-UBC-eGFP used for embryo microinjection were treated and purified in the same way as that for *in vitro* transcription templates. For microinjection, the purified p2IS-UBC-eGFP plasmids were mixed with different concentrations of NLS-I-SceI mRNA or included in the digestive reaction system of I-SceI endonuclease (NEB) as the substrate as previously described for fish transgenesis [31]. The I-SceI nuclease was stored at −80°C in 2 µL aliquots and added into the reaction system prior to microinjection as described [31], and its activity was confirmed by digestion of the plasmid p2IS-UBC-eGFP. To observe the localization of the injected DNA, two completely complementary 130 bp-long Cy3-labeled single strand DNA fragments containing two inversely flanking I-SceI recognition sequences at both ends were synthesized, denatured and annealed to be double-stranded, and then used for embryo cytoplasmic microinjection with NLS-I-SceI mRNA in the same way as transgene vector plasmids.

Microinjection was performed as described [33], except that the materials were injected into cytoplasm instead of pronuclear in this study. The mouse or porcine embryos subjected to microinjection were collected from mated female individuals and cultured as described [33], [21]. The porcine oocytes were collected from ovaries and subjected to *in vitro* maturation (IVM) as described [34]. The matured oocytes at metaphase of meiosis II (MII phase) with extruded first polar body were selected and subjected to microinjection post parthenogenetic activation by direct current electrical pulses (1.2 KV/cm, 30 µs, two times, 1 sec interval) as described [34]. The parthenogentically activated porcine oocytes (parthenogenetic embryos) were cultured as that for the collected porcine embryos.

The cultured embryos were observed under fluorescence microscopy or laser scanning confocal microscopy (LSCM, Zeiss LSM 780) to examine transgene expression or the localization of injected Cy3-labeled DNA fragments. To stain chromosomal DNAs, embryos were incubated in culture media containing 15 µg/mL Hoechst 33342(Sigma) for 30 min prior to microinjection and washed thoroughly in fresh media. To obtain transgenic founders, injected embryos were surgically transferred into oviducts of synchronized recipient female mice or sows as described [33], [21].

### Analysis of the presence of uncut I-SceI recognition site in embryos by polymerase chain reaction (PCR)

Total DNA samples were extracted from individual embryos by incubating each embryo in 10 µL of lysis buffer (KCl: 50 mM; MgCl2∶1.5 mM; Tris-Cl (pH8.0): 10 mM; Nonidet P-40∶0.5% (w/v); Tween-20∶0.5% (v/v); proteinase K: 100 µg/mL) at 65°C for 1 h. After heated at 95°C for 10 min to inactivate proteinase K, the lysate was used as template for PCR. A set of primer pair IS-site-F1/R1 (IS-site-F1∶5′-CCACTGACCTTTGGATGGTG-3′; IS-site-R1∶5′-TACCGCCTTTGAGTGAGCTG-3′; product size: 518 bp), of which the PCR product covered the I-SceI recognition sequence 3′ to the transgene cassette, was designed to detect the presence of uncut I-SceI site. Another primer pair set eGFP-F1/R1 (eGFP-F1∶5′-ACTGGAGAACTCGGTTTGTCGT-3; eGFP-R1∶5′-ACGGCCAGAATTTAGCGGAC -3′; product size: 453 bp) was used to detect the presence of eGFP CDS. The total DNA samples were further subjected to quantitative PCR (qPCR) analysis in a system based on SybrGreen qPCR Master Mix(2×) (ABI). The primer pair set for qPCR analysis of uncut I-SceI sites was IS-site-F2/R2 (IS-site-F2∶5′-AACTAGGGAACCCACTGCTT-3′; IS-site-R2∶5′-AACTAGGGAACCCACTGCTT-3′; product size: 171 bp), and that for qPCR of the eGFP CDS (the internal control) was eGFP-F2/R2 (eGFP-F2∶5′-CAGAAGAACGGCATCAAGGT-3′; eGFP-R2∶5′-TCTCGTTGGGGTCTTTGCT-3′; product size: 172 bp). Using the p2IS-UBC-eGFP plasmids diluted to different concentrations as standard samples, the qPCR analysis was performed in an absolute quantitation manner.

### Transgenic animal screen

Transgenic animals were screened by PCR and Southern blot assay. The primer pair set used for transgenic mouse screen by PCR was eGFP-F3/R3, of which the sequences were 5′-ATGGTGAGCAAGGGCGAGGA-3′ (eGFP-F3) and 5′-TGCCGTCCTCGATGTTGTGG-3′ (eGFP-R3), and the product size was 526 bp. The primer pair used for transgenic pig screen was eGFP-F1/R1 as described above. The probe for Southern blot assay was prepared by PCR using PCR DIG Probe Synthesis Kit (Roche) as described in the kit manual. The primer pair set used for probe preparation was Probe-DIG-F/R, of which the sequences were 5′-GCAGAAGAACGGCATCAAGGT-3′ (Probe-DIG-F) and 5′-TAGGGAGGGGGAAAGCGAA-3′ (Probe-DIG-R), which covered the junction region between eGFP CDS and the poly(A) signal sequence. Southern blot was performed using DIG-High Prime DNA Labeling and Detection Starter Kit II (Roche) as described in manual using genomic DNAs (>10 µg) completely digested by PstI. The *in vivo* green fluorescence in transgenic animals was detected using a GFP Macroscopy system (BLS, Hungarian) by exposure to blue excitation light with wave length of 460–495 nm and observed through a filter.

## Results

### The NLS-I-SceI molecule and transgene construct

**Figure 1:**
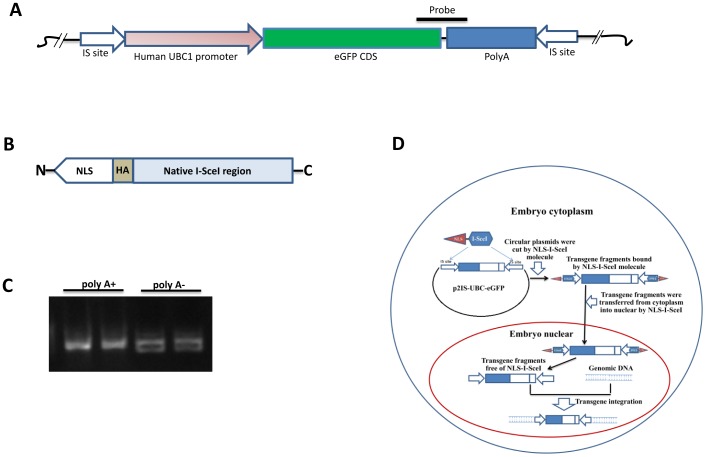
Transgene construct and the NLS-I-SceI molecule. A: The schematic structure of p2IS-UBC-eGFP vector. IS site: the inversely flanking I-SceI recognition sequence; the black bar indicates the position of the probe used for Southern blot assay. B: The schematic structure of NLS-I-SceI molecule. C: The *in vitro* transcribed NLS-I-SceI mRNA. polyA+: the mRNA with polyA tail; polyA-: the mRNA without polyA tail. D: The expected working principle of NLS-I-SceI-mediated transgenesis.

The NLS-I-SceI molecule consists of 3×SV40 NLS, an HA tag epitope and the native I-SceI molecule as shown in Fig. 1 B. 3×SV40 NLS is highly potent for nuclear localization, and HA tag epitope can be used to detect NLS-I-SceI molecule distribution in cells once it was expressed in cytoplasm. The NLS-I-SceI CDS was optimized for both porcine and murine codon usage preferences (the NLS-I-SceI CDS and amino acid sequence were shown in Fig. S1 and S2). High quality NLS-I-SceI mRNA was produced by T7 promoter-driven *in vitro* transcription using the linearized PCI-T7-NLS-I-SceI vector as templates (Fig. 1 C). The complete sequence of transgene vector p2IS-UBC-eGFP was shown in Fig. S3. In the transgene vector, a UBC promoter-driven eGFP expression cassette was flanked by two inversed I-SceI recognition sequences at both ends (Fig. 1 A). After cut by NLS-I-SceI molecule, the transgene vector plasmid was linearized, and the NLS-I-SceI protein was expected to be bound to the fragment containing transgene expression cassette, and thereby protect the transgene fragments from degradation and transfer the fragments from cytoplasm into nuclear (Fig. 1 D), for I-SceI protein exhibited high affinity in binding to the downstream cleavage product [36].

### The NLS-I-SceI molecule was capable of cutting circular transgene plasmids and transferring DNA fragments from cytoplasm into nuclear in mammalian embryos

**Figure 2:**
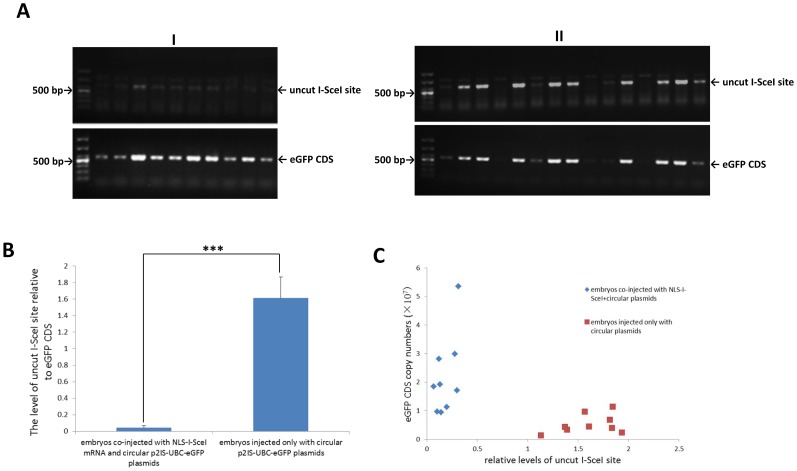
The NLS-I-SceI molecule was capable of cutting circular p2IS-UBC-eGFP plasmids in porcine parthernogenetic embryos. A: Detection of uncut I-SceI site and eGFP CDS by PCR in the embryos cytoplasmically injected with circular p2IS-UBC-eGFP plasmids plus NLS-I-SceI mRNA and only with circular p2IS-UBC-eGFP plasmids. I: embryos cytoplasmically injected with circular p2IS-UBC-eGFP plasmids plus NLS-I-SceI mRNA; II: embryos cytoplasmically injected only with circular p2IS-UBC-eGFP plasmids. B: The levels of uncut I-SceI site relative eGFP CDS detected by qPCR in the injected embryos. C: The eGFP CDS copy numbers in the injected embryos. *: statistical significance.

To investigate whether the NLS-I-SceI molecule was capable of cutting the I-SceI recognition sequence-containing circular plasmids in mammalian embryos, total DNAs were extracted from single porcine parthenogenetic blastocysts developed from oocytes co-injected with NLS-I-SceI mRNAs and the circular p2IS-UBC-eGFP plasmids (30 ng/µL each), and subjected to PCR analysis to assess the extent to which the circular plasmids were digested. The uncut I-SceI site was quantitatively detected by qPCR using a primer pair covering the I-SceI recognition sequence 3′ to the transgene expression cassette, and the eGFP CDS detected as internal control. Prior to qPCR, a qualitative PCR was performed to confirm the existence of plasmids in embryos. As shown in Fig. 2 A, in the embryos co-injected with NLS-I-SceI mRNA and the circular transgene plasmids, the band intensities of PCR products covering I-SceI site were remarkably lower than those of eGFP CDS. In contrast, in embryos injected only with circular plasmids, the uncut I-SceI site and eGFP CDS were simultaneously detected or not in these samples (Fig. 2 A), and the band intensities of PCR products covering I-SceI site were comparable to those of eGFP CDS (Fig. 2 A), indicating that in these embryos the levels of uncut I-SceI site and eGFP CDS were comparable and varied proportionally. The samples with expected PCR products were subjected to qPCR analysis, of which the Amplification Plots, Melt Curves and Standard Curves were shown in Fig. S4. The qPCR data further showed that the levels of uncut I-SceI site relative to eGFP CDS in embryos co-injected with NLS-I-SceI mRNA and circular plasmids were largely lower than those in embryos injected only with circular plasmids (P<0.001, Fig. 2 B), indicating that the NLS-I-SceI molecule produced from mRNAs in mammalian embryos was bio-active and capable of cutting circular plasmids. Moreover, these data further demonstrated that in the embryos co-injected with NLS-I-SceI mRNA and circular plasmids, although the relative levels of uncut I-SceI site were much lower, the eGFP CDS copy numbers were remarkably higher than those in embryos injected only with circular plasmids (P<0.001, Fig. 2 C), suggesting that the linearized transgene DNA fragments were protected from degradation by NLS-I-SceI molecule after plasmids were cut at I-SceI sites.

**Figure 3:**
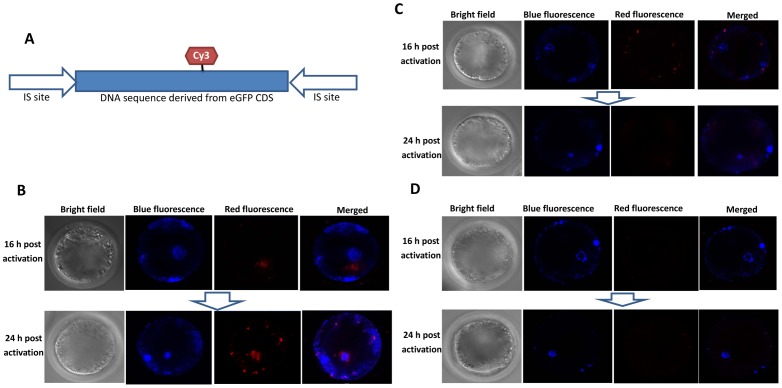
Transfer of DNA fragments from cytoplasm into nuclear by NLS-I-SceI molecule in porcine parthernogenetic embryos. The activated porcine MII oocytes (1-cell parthernogenetic embryos) stained with Hoest33342 were cytoplasmically injected with Cy3-labelled DNA fragments plus NLS-I-SceI mRNA, and the localization of DNA fragments were observed under LSCM at 16 and 24 h post microinjection respectively. In control groups, the embryos were injected with Cy3-DNA fragments included into the native I-SceI endonuclease digestive reaction system or only with Cy3-DNA fragments. A: The structure of Cy3-labeled DNA fragments. B: The localization of Cy3-DNA fragments co-injected with NLS-I-SceI mRNA. C: The localization of Cy3-DNA fragments co-injected with the native I-SceI nuclease. D: The localization of Cy3-DNA fragments injected alone. Red fluorescence: the Cy3-DNA fragments; Blue fluorescence: the chromosomal DNAs.

To display the localization of injected DNAs in living embryos, Cy3-labeled double-stranded DNA (Cy3-DNA) fragments containing two inversely flanking I-SceI recognition sequences at both ends, of which the schematic structure was shown in Fig. 3 A and sequence Fig. S5, were co-injected with NLS-I-SceI mRNA into the cytoplasm of activated porcine MII oocytes (parthenogenetic embryos) of which the chromosomal DNAs were stained with Hoechst 33342 prior to microinjecction. The injected embryos were cultured and observed under LSCM at 16 and 24 h post activation. In control groups, the embryos were injected with only Cy3-DNA fragments or Cy3-DNA fragments included in the native I-SceI endonuclease digestive reaction system as that for I-SceI-mediated transgenesis in fish. At 16 h post activation, in the embryos co-injected with NLS-I-SceI mRNAs and Cy3-DNAs, of which the chromosomes were in a relaxed state and loosely assembled suggesting that meiosis was proceeding to the telophase and the nuclear was under construction (Fig. 3 B), the Cy3-DNA fragments (red fluorescence) were found to be clustered and located near to the chromosomes (blue fluorescence) (Fig. 3 B). At 24 h post activation, in the embryos co-injected with NLS-I-SceI mRNAs and Cy3-DNAs, the blue fluorescence was concentrated suggesting that chromosomes were compactly aggregated, meiosis completed and nuclear was constructed, and the clustered Cy3-DNA fragments were observed to be completely co-localized with chromosomes (Fig. 3 B), indicating that the Cy3-DNA fragments were transferred into nuclear. In contrast, in the embryos of control groups, the red fluorescence was scattered and extremely weak, and no Cy3-DNAs were observed to be clustered, located closely to or co-localized with the chromosomes at 16 h or 24 h post activation (Fig. 3 C, D), suggesting that the Cy3-DNA fragments were diffusely distributed in cytoplasm or degraded. These data provided a direct demonstration that the NLS-I-SceI molecule was capable of transferring DNA fragments from cytoplasm into nuclear in mammalian embryos, and this transfer process was co-incident with the process of nuclear formation during meiosis (or mitosis) of embryos, while the native I-SceI molecule was not, being consistent with previously reported data for the native I-SceI-mediated transgenesis in fish embryos [30].

### NLS-I-SceI molecule was capable of facilitating transgenesis in mammalian embryos via cytoplasmic microinjection

**Figure 4:**
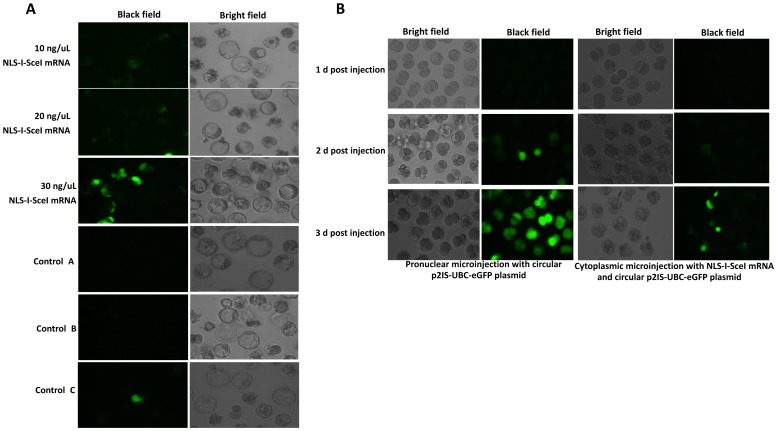
Transgene expression in cytoplasmically injected mouse embryos. A: Mouse eggs cytoplasmically injected with 30 ng/µL of circular p2IS-UBC-eGFP plasmids plus NLS-I-SceI mRNAs at different concentratiions. Controls A-C were the control groups injected with 30 ng/µL circular plasmids included into the native I-SceI endonuclease digestive reaction system (control A), linearized plasmids (control B) or circular plasmids (control C). B: The dynamics of transgene expression in embryos subjected to cytoplasmic microinjection with circular p2IS-UBC-eGFP plasmids plus NLS-I-SceI mRNA or pronuclear microinjection only with circular p2IS-UBC-eGFP plasmids.

To investigate whether NLS-I-SceI molecule was capable of mediating transgenesis and resulting in transgene expression in early mammalian embryos, mouse eggs were subjected to cytoplasmic microinjection with the mixture of NLS-I-SceI mRNA and circular transgene plasmid p2IS-UBC-eGFP, for mouse eggs have visible pronuclear and the materials can be confirmed to be injected into cytoplasm. With a given plasmid concentration (30 ng/µL), NLS-I-SceI mRNAs at different concentrations (10, 20 and 30 ng/µL) were co-injected with circular transgene plasmids into cytoplasm of 1-cell mouse eggs, and green fluorescence in the blastocysts developed from injected eggs were observed and counted at 5 d post injection. To avoid cellular lysis after injection, a very small volume (about 5 pL) of solution, which was less than that for pronuclear microinjection, was injected into embryo cytoplasm. Results showed that the NLS-I-SceI molecule mediated transgenesis in a dose-dependent manner (Fig. 4 A). In the group injected with 30 ng/µL of NLS-I-SceI mRNA, the fluorescence intensity was significantly higher than those in groups injected with 10 and 20 ng/µL of NLS-I-SceI mRNA (Fig. 4 A). In contrast, in the group injected with 30 ng/µL of circular p2IS-UBC-eGFP plasmid included in the native I-SceI endonuclease digestive reaction system, no fluorescent blastocysts were observed (Fig. 4 A), indicating that without the added NLS signal, the native I-SceI molecule was not capable of facilitating transgenesis and further resulting in transgene expression in mouse embryos. In the groups cytoplasmically injected only with circular or linearized plasmids at the same concentration, no fluorescence was observed in the derived blastocysts either, although fluorescence was observed in a few developmentally arrested embryos in the circular plasmid injection group (Fig. 4 A). In all the groups, the blastocyst development rates (blastocysts/cleaved eggs) were comparable to the untreated group (data not shown), suggesting that the injected materials did not interfere with *in vitro* development once embryos survived the microinjection process. The dynamics of transgene expression in the embryos cytoplasmically co-injected with NLS-I-SceI mRNA and circular transgene plasmid was similar to that in embryos subjected to pronuclear microinjection only with circular transgene plasmid, although the fluorescence intensity in the cytoplasmic injection group was lower (Fig. 4 B), suggesting that the transgene fragments delivered into cytoplasm were transferred into pronuclear by NLS-I-SceI molecule as early as embryo cleavage started, which was consistent with the results of LSCM observation. The lower fluorescence intensity may be due to the less copies of transgene fragment in pronuclear transferred from cytoplasm by NLS-I-SceI molecule compared to those of transgene fragment directly delivered into pronuclear by microinjection.

**Figure 5:**
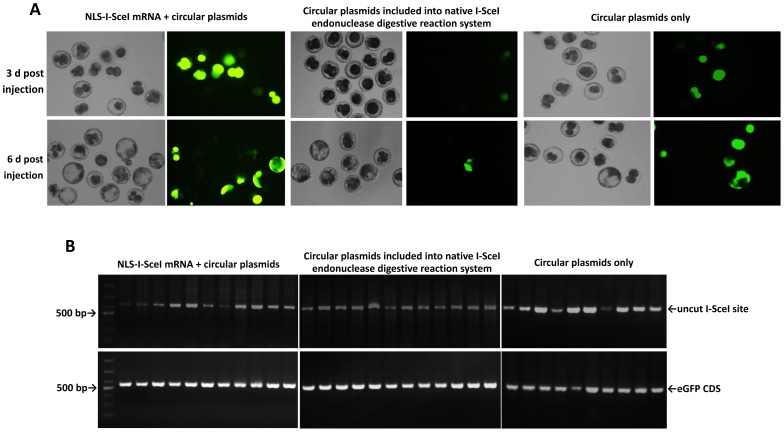
Transgene expression and detection of uncut I-SceI site and eGFP CDS by PCR in cytoplasmically injected porcine embryos. A: Transgene expression in the porcine embryos cytoplasmically injected with circular plasmids (p2IS-UBC-eGFP) plus NLS-I-SceI mRNA, circular plasmids included into the native I-SceI nuclease digestive reaction system and circular plasmids only. B: Detection of uncut I-SceI site and eGFP CDS by PCR in the cytoplasmically injected porcine embryos as described in A.

**Figure 6:**
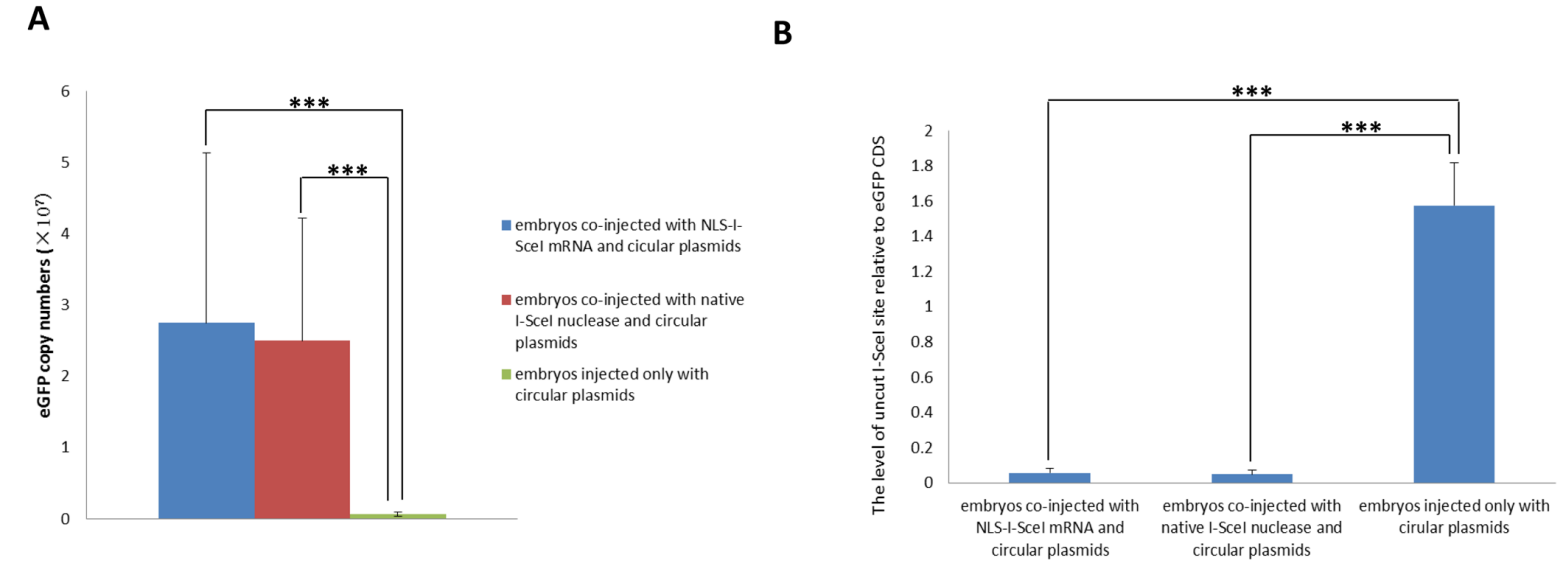
Quantitative analysis of uncut I-SceI site and eGFP CDS by qPCR in cytoplasmically injected porcine embryos. A: The eGFP CDS copy numbers in the cytoplasmically injected porcine embryos as described in Fig. 5. B: The uncut I-SceI site levels relative to eGFP CDS in the cytoplasmically injected porcine embryos as described in Fig. 5. *: statistical significance.

To address whether NLS-I-SceI molecule was capable of mediating transgenesis in mammalian embryos of species other than mice, 1- or 2-cell porcine eggs surgically collected from mated sows were subjected to cytoplasmic co-injection with NLS-I-SceI mRNAs and circular transgene plasmids (30 ng/µL each), for pig is a typical mammalian species of which the pronuclear is usually invisible and refractory to pronuclear microinjection. Porcine eggs have a relatively larger size and are much more tolerant to cytoplasmic microinjection compared to mouse eggs, and a much larger volume (40–60 pL) of solution, which contained 1.2–1.8 pg of transgene plasmids and NLS-I-SceI mRNAs respectively, was injected into cytoplasm. As shown in Fig. 5 A, in the porcine embryos derived from eggs co-injected with NLS-I-SceI mRNA and circular p2IS-UBC-eGFP plasmids, strong fluorescence was observed on 3 d post injection, and the majority of derived blastocysts exhibited strong fluorescence on 6 d post injection. In contrast, in the embryos injected with circular p2IS-UBC-eGFP plasmids (30 ng/µL) included into the native I-SceI endonuclease digestive reaction system, only weak fluorescence was observed in a few embryos on 3 d post injection, and on 6 d, no fluorescence was observed in the derived blastocysts, although fluorescence was observed in a few developmentally arrested embryos (Fig. 5 A). This different fluorescence was not because of the difference in eGFP CDS copy numbers, for the eGFP CDS was readily detected in all the injected embryos (Fig. 5 B), and the eGFP CDS copy numbers in the embryos injected with circular plasmids plus NLS-I-SceI mRNA were comparable to those in embryos injected with circular plasmids at the same concentration included into the native I-SceI endonuclease digestive reaction system (P>0.1, Fig. 6 A), which were much higher than those in embryos injected only with circular plasmids (P<0.001, Fig. 6 A). The uncut I-SceI site was detected in the injected embryos (Fig. 5 B), and its levels relative to eGFP CDS were also comparable between the two groups injected with circular plasmids plus NLS-I-SceI mRNA and native I-SceI nuclease (P>0.1, Fig. 6 B), but were significantly lower than those of the group injected only with circular plasmids (P<0.001, Fig. 6 B), suggesting that the NLS-I-SceI molecule derived from mRNA cut circular plasmids to a similar degree to the native I-SceI nuclease in porcine embryos. The presence of uncut I-SceI site indicated that there existed residual circular plasmids in the injected embryos, which may be a reason for the fluorescence in the few porcine embryos injected with the circular plasmids included into the native I-SceI endonuclease digestive reaction system. The circular plasmids were resistant to endogenous nuclease and could be passively diffused into the nuclear during embryo cleavage as indicated by a previous report [36]. Consistently, in this work, the porcine embryos injected only with circular plasmids also exhibited fluorescence (Fig. 5 A), while those injected with linearized plasmids did not (data not shown). However, the circular plasmid rarely results in transgene integration in mammalian embryos even introduced directly into pronuclear in large amounts [27], [36]. Totally, these data indicated that the NLS-I-SceI molecule was capable of efficiently facilitating transgenesis in porcine embryos, while the native I-SceI molecule was not.

### The transgenesis mediated by NLS-I-SceI molecule in mammalian embryos efficiently resulted in transgenic animals

**Figure 7:**
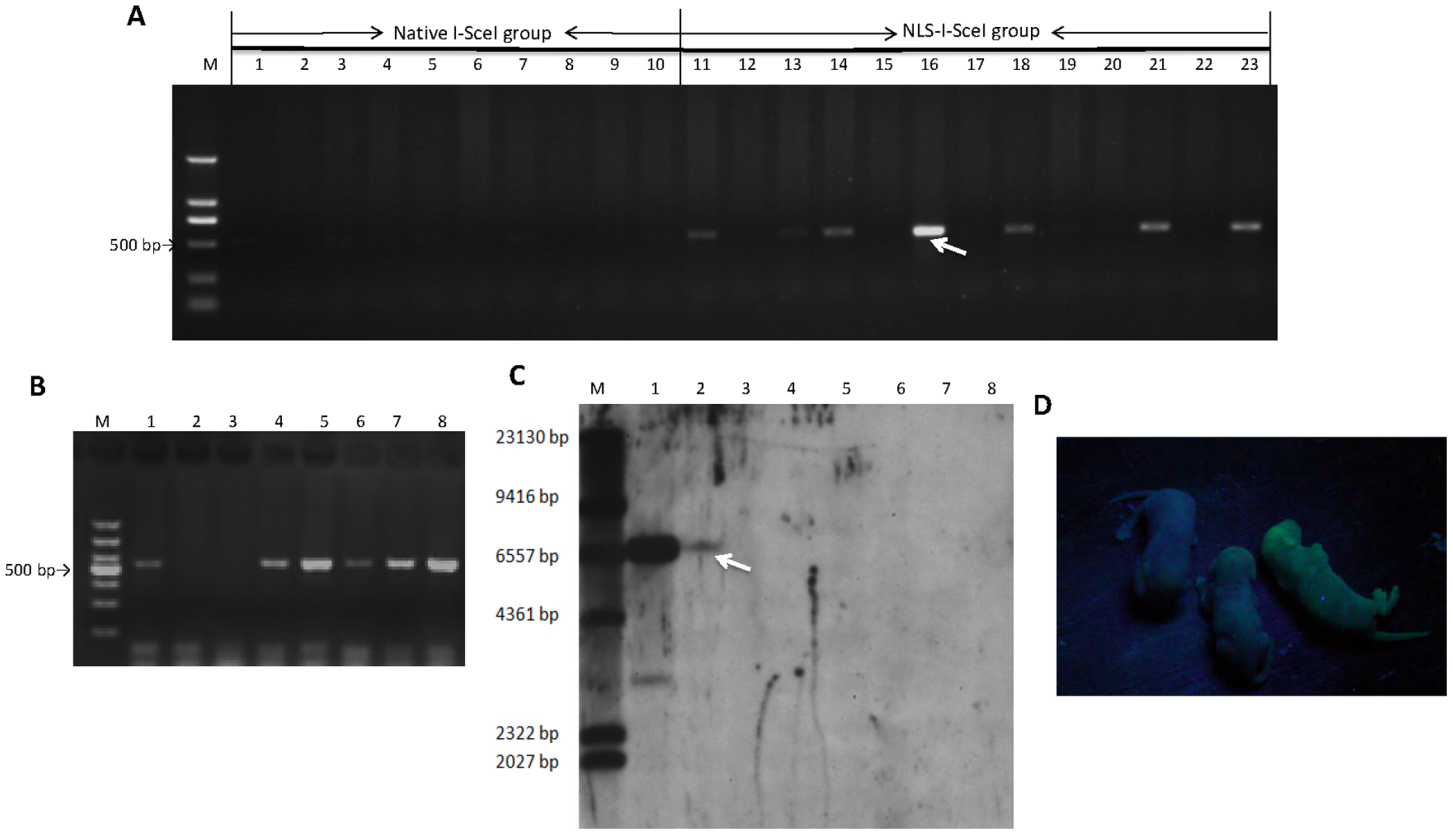
Genetic screen of transgenic mice derived from cytoplasmically microinjected eggs. A: Screen of transgenic founder mice by PCR. M: DL2000 DNA marker; 1–10: the founder mice derived from cytoplasmic microinjection with circular p2IS-UBC-eGFP plasmids (30 ng/µL) included into the native I-SceI nuclease digestive reaction system; 11–23: The founder mice derived from cytoplasmic microinjection with circular p2IS-UBC-eGFP plasmids plus NLS-I-SceI mRNA (30 ng/µL each). B: Screen of transgenic individuals of F1 offspring derived from transgenic founder mice by PCR. M: DNA marker; 1–8: Genomic DNA samples of F1 individuals. C: Genetic screen of transgenic founder mice by Southern blot assay. M: DNA molecular weight marker II; 1: plasmids; 2–7: genomic DNA samples of founder mice; 8: negative control (wild-type mouse genomic DNA). D: The transgenic mice exhibiting *in vivo* fluorescence derived from breeding between transgenic individuals over three consecutive generations. The arrow indicates the founder mouse detected to be transgenic by both Southern blot and PCR screen.

To answer whether the NLS-I-SceI-mediated transgenesis in mammalian embryos via cytoplasmic microinjection was able to result in transgenic animals, 411 fertilized mouse eggs were collected from nine super-ovulated and mated female mice, and 330 eggs with visible pronuclear were selected and randomly and equally divided into two groups. One group was subjected to cytoplasmic microinjection with the mixture of NLS-I-SceI mRNA and circular transgene plasmids (30 ng/µL each), and the other group (control) injected with circular transgene plasmid (30 ng/µL) included into the native I-SceI endonuclease digestive reaction system as described above. 116 eggs which survived the microinjection process and cleaved the next day were transferred into 4 surrogate mice. Totally, 23 founder pups were born, of which 10 pups were derived from eggs of control group, and 13 pups from eggs co-injected with NLS-I-SceI mRNA and circular plasmids. As shown in Fig. 7 A, in the founders derived from eggs co-injected with NLS-I-SceI mRNA and circular plasmids, 6 pups were detected to be transgenic by PCR, while in the control group no transgenic pub was detected. The transgenic rate in founders of NLS-I-SceI-mediated transgenesis group was 46.2% (6/13), and the transgenesis efficiency (transgenic founders/transferred eggs) was 10.7% (6/56), which were both higher than the data for pronuclear microinjection in our lab (unpublished). However, the survival rate of cytoplasmically microinjected mouse eggs (35.2% (116/330)) was remarkably lower than that of eggs subjected to pronuclear microinjection in our lab (usually 50%), indicating that mouse eggs were more vulnerable to cytoplasmic microinjection than to pronuclear microinjection. To test the germline transmission competence of transgene, the transgenic founder mouse with the strongest PCR product band were mated with wild-type mice, and transgenic individuals were detected from the resulted offspring (Fig. 7 B). *In vivo* fluorescence was not observed in the transgenic founder mice or the transgenic individuals of F1 offspring, and transgene integration was detected by Southern blot only in one founder mouse (Fig. 7 C). However, the *in vivo* fluorescence was observed after the transgenes were enriched by mating between transgenic individuals consecutively over at least three generations (Fig. 7 D), indicating that the NLS-I-SceI-mediated transgenesis did resulted in transgene integration in mouse genome although not detected by Southern blot assay in most founders. These results indicated that the NLS-I-SceI-mediated transgenesis was capable of resulting in transgenic mice, while the native I-SceI nuclease was not.

**Figure 8:**
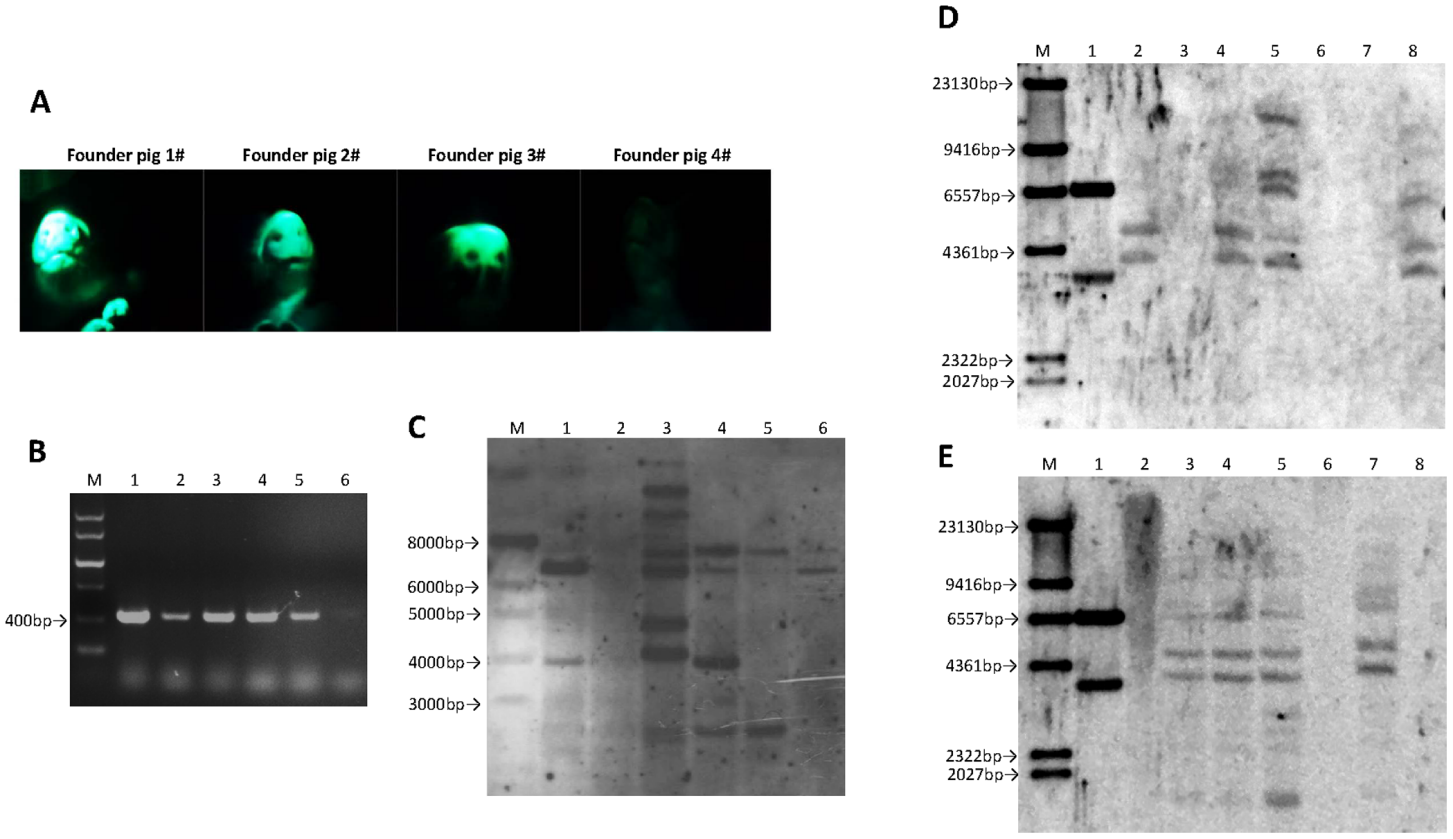
Genetic screen of transgenic pigs derived from embryos cytoplasmically microinjected with circular p2IS-UBC-eGFP plasmids plus NLS-I-SceI mRNA. A: *In vivo* fluorescence in founder pigs. B: Screen of transgenic founder pigs by PCR. M: DNA marker; 1–4: genomic DNA samples of 1–4# founder pigs; 5: positive controls (wild-type pig genomic DNAs containing p2IS-UBC-eGFP plasmids); 6: negative control (wild-type pig genomic DNA). C: Southern blot assay of transgenic founder pigs. M: DNA marker (1KB DNA Ladder); 1: positive control (plasmids); 2: wild-type pig genomic DNA as negative control; 3–6∶1–4# founder pigs. D: Southern blot analysis of F1 offspring individuals derived from founder pig 1#. M: DNA molecular weight marker II; 1: plasmid as positive control; 2–8: the F1 offspring individuals. E: Southern blot analysis of genomic DNAs extracted from different organs of founder pig 1#. M: DNA molecular weight marker II; 1: positive control (plasmids); 2: skin; 3: heart; 4: liver; 5: spleen; 6: lung; 7: kidney; 8: wild-type pig genomic DNA as negative control.

To further test whether the NLS-I-SceI-mediated transgenesis would result in transgenic animals of species other than mice, 36 porcine eggs at 1- or 2-cell stage surgically collected from mated sows were subjected to cytoplasmic co-injection with NLS-I-SceI mRNA and the circular p2IS-UBC-eGFP plasmids, and then transferred into two synchronized surrogate sows. One recipient was pregnant and four piglets were born. The *in vivo* fluorescence was observed in three of the four founder pigs (Fig. 8 A). However, all of the founder pigs were detected to be transgenic by PCR screen and transgene integration was confirmed by Southern blot assay (Fig. 8 B, C). The lack of *in vivo* fluorescence in one transgenic founder pig (4#) may be due to the low copy number of integrated transgenes as indicated by the Southern blot data (Fig. 8 C). The founder pig with the strongest fluorescence (1#) was mated with wild-type pig to test the germline transmission competence of transgenes. As shown in Fig. 8 D, in the seven individuals of F1 offspring, four were detected to be transgenic by Southern blot, indicating that the transgenes were capable of germline transmission. After gemline transmission was confirmed, the founder pig (1#) was sacrificed due to disease related to respiratory system infection, and genomic DNA samples of different organs were subjected to Southern blot assay. As shown in Fig. 8 E, transgene was detected in all the organs except skin and lung in a similar band distribution pattern. However, the failure to detect transgene in these two organs was due to the experimental procedure but not to the lack of transgene integration, for the genomic DNAs of the two organs were not thoroughly digested and separated in gel electrophoresis as a result before DNA was transferred to membrane (Fig. S6 A), and transgene was finally detected in these two organs with a similar band distribution pattern by a repeated Southern blot assay after the genomic DNAs were completely digested (Fig. S6 B, C), suggesting that this founder pig was not transgenically mosaic and transgene integration occurred at a very early stage of embryo development. The death of the founder pig was not due to transgenesis, for some wild-type pigs in the farm also died of the same disease at that time. The rest transgenic pigs, including the offspring of the dead founder pig, kept healthy. These results demonstrated that the NLS-I-SceI-mediated transgenesis in mammalian embryos was capable of efficiently resulting in transgenic animals with germline transmission competence, especially in species other than mice which was refractory to embryo pronuclear microinjection but exhibited higher tolerance to embryo cytoplasmic microinjection.

## Discussion

Embryo microinjection is a simple and reproducible method for mammalian transgenesis, however the dependence on visible pronuclear largely limits its application to mammalian species other than mice, especially those large animal species of which the pronuclear is usually invisible. Currently, transgenisis via embryo cytoplasmic microinjection has achieved limited success in mammalian species. Page et al (2005) produced transgenic mice using Polylysine/DNA mixture by cytoplasmic microinjection of eggs, however the transgenic rate (born transgenic pups/transferred embryos) was much lower than that of pronuclear microinjection (12.8% vs 21.7%) [37]. Garrels et al (2011) efficiently produced transgenic pigs by Sleeping Beauty (SB) transposon-mediated transgenesis via embryo cytoplasmic microinjection with circular plasmids of SB transposon-based transgene vector and SB tranposase expression vector, and the transgenic rate of founder pigs was as high as 47.3% [23]. Nonetheless, transposons are mobile genetic elements and transgenic organisms derived from transposon-mediated transgenesis would be of bio-safety concerns. Recently, Wilson et al (2013) has described a sophisticated system termed intracellular electroporetic nanoinjection (IEN) to propel transgene fragments from cytoplasm into pronuclear, however this method required additional complicated equipment and experimental skills besides conventional microinjection, and more importantly, the transgenesis efficiency of IEN system was not higher (actually slightly lower) than that of pronuclear microinjection [38].

I-SceI has been effectively used to facilitate transgenesis in fish eggs for several years. Because only plasmid vectors containing I-SceI recognition sequences are involved in the I-SceI-mediated transgenesis, regarding the transgenesis process and the application of the resulted transgenic organisms, the I-SceI-mediated transgenesis is of minimal bio-safety concerns. In this work, we efficiently generated transgenic mammals (pigs and mice) simply by co-injecting circular transgene vector plasmids containing I-SceI recognition sequences and the mRNAs coding NLS-I-SceI molecule into embryo cytoplasm. As far as we know, this is the first report for efficient generation of transgenic mammals via embryo cytoplasmic microinjection using the I-SceI molecule.

Our work demonstrated that the native I-SceI molecule was not capable of efficiently facilitating transgenesis in mammalian embryos as it did in fish eggs, which may be due to the much smaller size of mammalian embryos compared to that of fish eggs and much less plasmid copies that can be delivered into mammalian embryos as a result. In contrast, the NLS-I-SceI molecule, which contains mammalian NLS sequence at its N-terminal, was shown to be capable of cutting transgene fragments off from circular plasmids, protecting transgene fragments from degradation and efficiently facilitating transgenesis in both mouse and porcine embryos, indicating that the artificially added mammalian NLS signal largely promoted the efficacy of I-SceI-mediated transgenesis. The ability of NLS-I-SceI molecule to facilitate transgenesis in mammalian embryos was directly demonstrated by the localization of Cy3-labeled DNA fragments containing inversely flanking I-SceI cutting sites at both ends which were co-injected with NLS-I-SceI mRNA into the cytoplasm of porcine pathenogenically activated oocytes at MII stage (parthenogenetic embryos). The reason for the use of porcine MII oocytes was that the nuclear was breakdown at this stage and to be constructed upon activation, providing a time window to observe the localization of DNA fragments during the process of mammalian pronuclear construction, and that in addition, the lack of nuclear excluded the probability that materials happened to be injected into nuclear by chance due to the invisibility of pronuclear. Data showed that only the DNA fragments co-injected with NLS-I-SceI molecule were clustered and co-localized with chromosomes in parthenogenetic porcine embryos, while those co-injected with the native I-SceI molecule were diffusely distributed in the cytoplasm and not clustered or co-localized with chromosomes, indicating that the NLS-I-SceI was capable of transferring DNA fragments from cytoplasm into nuclear, while the native I-SceI molecule was not, and the transferring process was co-incident with the procedure of nuclear formation. These results were consistent with the observation that the porcine blastocysts developed from eggs co-injected with NLS-I-SceI mRNA and circular transgene plasmids p2IS-UBC-eGFP exhibited strong fluorescence, while those co-injected with the native I-SceI nuclease and circular transgene plasmids at the same concentration did not, although the eGFP CDS was detected at similar levels in these embryos, suggesting that although the transgene fragments were efficiently cut off from circular plasmids and protected from degradation by the native I-SceI nuclease in porcine embryos, the transgene fragments were not efficiently translocated from cytoplasm into nuclear by this molecule to result in expression. The efficient production of transgenic mice and pigs and the germline transmission competence of the resulted transgenic animals further confirmed that the NLS-I-SceI molecule can be used as a potent tool to facilitate mammalian transgenesis.

The NLS-I-SceI-mediated transgenesis resulted in random integration in mammalian genome. With the advent of powerful EENs such as ZFN, TALEN and CRIPR/Cas9 system, the NLS-I-SceI molecule can be used in combination with EENs to facilitate targeted transgene integration into mammalian embryo genomes. Recently, it has been reported that *in vivo* cleavage of circular plasmids by EENs effectively facilitated targeted integration of transgenes into the DSBs created by the same or another EEN molecule through none-homologous end joining (NHEJ) mechanism in the genomes of mammalian somatic cells [39], [40]. However, the ability of the EENs to bind to the cleaved DNAs and further transfer DNA fragments from cytoplasm into nuclear of mammalian embryos remains to be investigated, although these molecules have NLS signal. More recently, Cas9-sgRNA complex was shown to be capable of binding cleaved DNA with high affinity, however the both ends of cleaved DNA were tightly bound to Cas9-sgRNA complex and the cleaved circular plasmids were still in circular form [41], which would hinder transgene integration. On this basis, considering the confirmed ability of NLS-I-SceI molecule to cut transgene fragments off from circular transgene plasmids, protect transgene fragments from degradation and transfer transgene fragments from cytoplasm into nuclear in mammalian embryos, NLS-I-SceI molecule can be used in combination with EENs to facilitate targeted transgene integration into mammalian embryo genomes, and thereby a simple, efficient and species-neutral technology for targeted transgenesis in mammalian animals can be established, especially for large mammalian species such as pig, cattle and none-human primates.

In this work, we found that the native I-SceI molecule without mammalian NLS signal did not efficiently facilitate transgenesis in mouse or porcine embryos. However, Bevacqua et al (2013) recently reported that the native I-SceI-mediated transgenesis resulted in transgenic eGFP expression in bovine blastocysts derived from *in vitro* fertilization [42]. This inconsistency may be partly due to the much higher concentration (50 ng/µL) of circular transgene plasmids used in this study compared to that in our work (30 ng/µL). Such a high concentration may result in the presence of more uncut circular plasmids in embryos, which were resistant to degradation in cells and can be passively diffused into nuclear during embryo cleavage as suggested by a previous report [36] and our data in this work. However, circular plasmids rarely integrated into genome even directly delivered into pronuclear in a large amount, and no transgenic cattle was produced in this report either. Besides, the fluorescence in bovine blastocysts resulting from the native I-SceI-mediated transgenesis with plasmids of a natural promoter (Pax6)-driven eGFP expression vector was rather weak, which was comparable to that in the few fluorescent porcine embryos co-injected with the native I-SceI nuclease and circular p2IS-UBC-eGFP plasmids in this study. The moderately stronger fluorescence, of which the intensity was remarkably lower compared to that in the porcine embryos co-injected with NLS-I-SceI mRNAs and p2IS-UBC-eGFP plasmids in our work, resulted from transgenesis with another artificially synthesized strong promoter(CAG)-driven eGFP expression vector plasmids, suggesting that the relatively stronger fluorescence was due to the much higher activity of the CAG promoter, but not to the more transgene copies in nuclear, and the native I-SceI molecule did not actively or efficiently transfer transgene fragments from cytoplasm into nuclear in the *in vitro* fertilized bovine embryos either.

Because the circular DNA plasmids can be passively diffused into nuclear during embryo cleavage and the NLS-I-SceI molecule is nuclear-localized, we can’t exclude the possibility that the efficient NLS-I-SceI-mediated transgenesis in mammalian embryos was partly derived from *in situ* cleavage of circular plasmids by NLS-I-SceI molecule in nuclear. *In situ* cleavage of circular transgene plasmids in cells was shown to protect transgene fragments from degradation and facilitate transgene integration as a result [39], [40]. On this basis, considering that cytoplasmic microinjection with circular bacterial artificial vector (BAC) plasmids also resulted in transgene expression in mammalian embryos, suggesting that circular BAC plasmids can be passively diffused into nuclear once introduced into cytoplasm of embryos [35], the NLS-I-SceI molecule can be used to facilitate BAC transgenesis in mammalian embryos only if the I-SceI recognition sequences were included in BAC vectors.

In summary, this work demonstrated that the NLS-I-SceI molecule was capable of efficiently facilitating mammalian transgenesis, and using this molecule, a simple and efficient general transgenesis technology with minimal bio-safety concerns can be established for mammals. For fully validating this method, a transgenic animal model with exclusive characteristics can be generated via NLS-I-SceI-mediated transgenesis as a quality control, such as the transgenic pig model for human Huntington’s disease exhibiting apoptosis in brain neurons similar to human that is not observed in murine models harboring the same transgene [43]. In addition, to fully characterize this technology, the variation of transgene integration sites can be investigated in the future when more transgenic individuals were derived from NLS-I-SceI-mediated transgenesis.

## Supporting Information

Figure S1 **The coding sequence of NLS-I-SceI molecule.** The codon usage was optimized for both mice and pigs on the basis that possible splice sites were excluded.
(TIF)

Figure S2 **The amino acid sequence of NLS-I-SceI molecule.**
(TIF)

Figure S3 **The sequence of p2IS-UBC-eGFP vector.** The bold and underlined sequences are inversely flanking I-SceI recognition sequences, and the bold sequence in green is the eGFP CDS.
(TIF)

Figure S4 **The Amplification Plots, Melt Curves and Standard Curves for qPCR of the uncut I-SceI site and eGFP CDS.**
(TIF)

Figure S5 **The sequence of the Cy3-labeled DNA fragment.** The underlined sequences are the inversely flanking I-SceI recognition sequences, and the bold base in red is the one where the Cy3 fluorophore is linked.
(TIF)

Figure S6 **Repeated Southern blot analysis of transgene integration in the skin and lung of transgenic founder pig 1#.** The genomic DNA samples of the skin and lung of founder pig1# were not thoroughly digested with PstI endonuclease in the first Southern blot assay (A). In the repeated Southern blot analysis, the same genomic DNA samples were completely digested as indicated by gel electrophoresis (B), and then transgene integration was detected by Southern blot in the two organs (C). M: DNA marker (1 Kb ladder in gel electrophoresis, and DNA molecular weight marker II in Southern blot assay).
(TIF)
