# Supplementary figures and images for: The Meganuclease I-SceI Containing Nuclear Localization Signal (NLS-I-SceI) Efficiently Mediated Mammalian Germline Transgenesis via Embryo Cytoplasmic Microinjection
Source: PLoS One. 2014 Sep 24;9(9):e108347. doi: 10.1371/journal.pone.0108347 (PMC4177210; doi:10.1371/journal.pone.0108347)

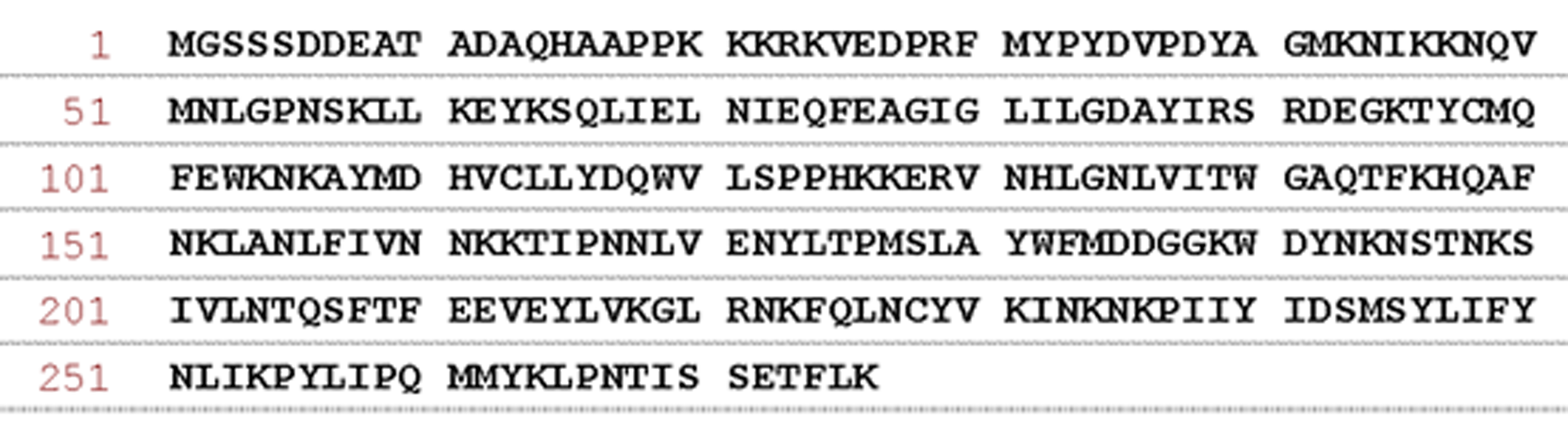

Supplement: Figure S2 — The amino acid sequence of NLS-I-SceI molecule. (TIF) [file pone.0108347.s002.tif]

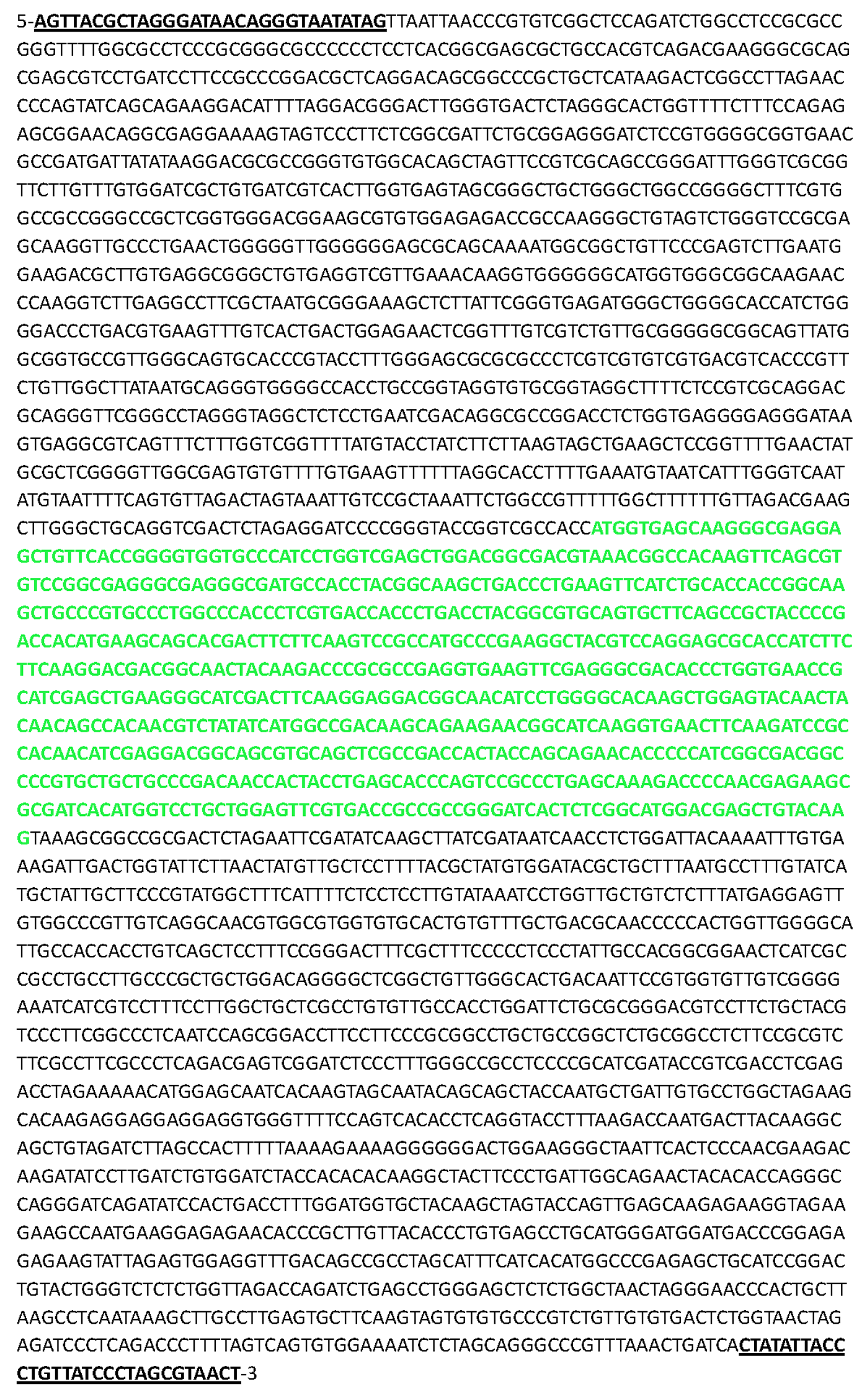

Supplement: Figure S3 — The sequence of p2IS-UBC-eGFP vector. The bold and underlined sequences are inversely flanking I-SceI recognition sequences, and the bold sequence in green is the eGFP CDS. (TIF) [file pone.0108347.s003.tif]

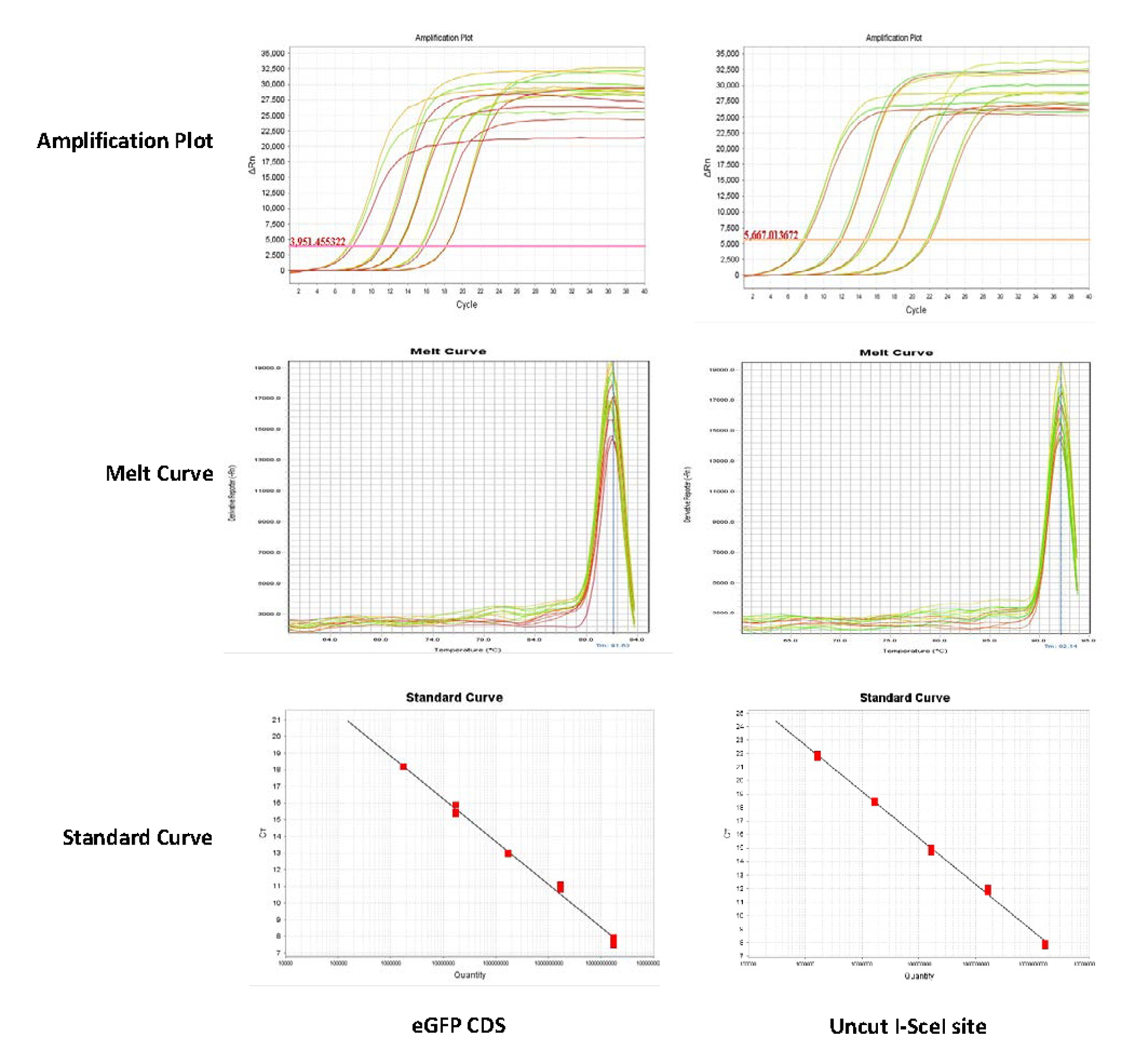

Supplement: Figure S4 — The Amplification Plots, Melt Curves and Standard Curves for qPCR of the uncut I-SceI site and eGFP CDS. (TIF) [file pone.0108347.s004.tif]

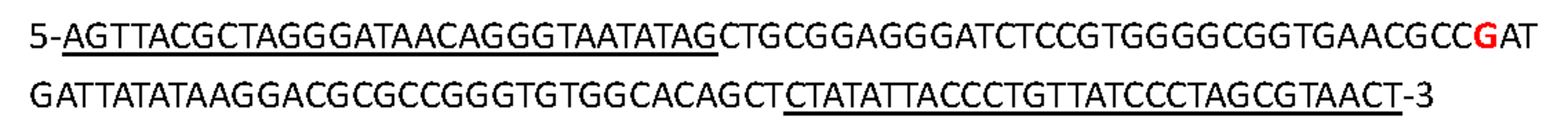

Supplement: Figure S5 — The sequence of the Cy3-labeled DNA fragment. The underlined sequences are the inversely flanking I-SceI recognition sequences, and the bold base in red is the one where the Cy3 fluorophore is linked. (TIF) [file pone.0108347.s005.tif]

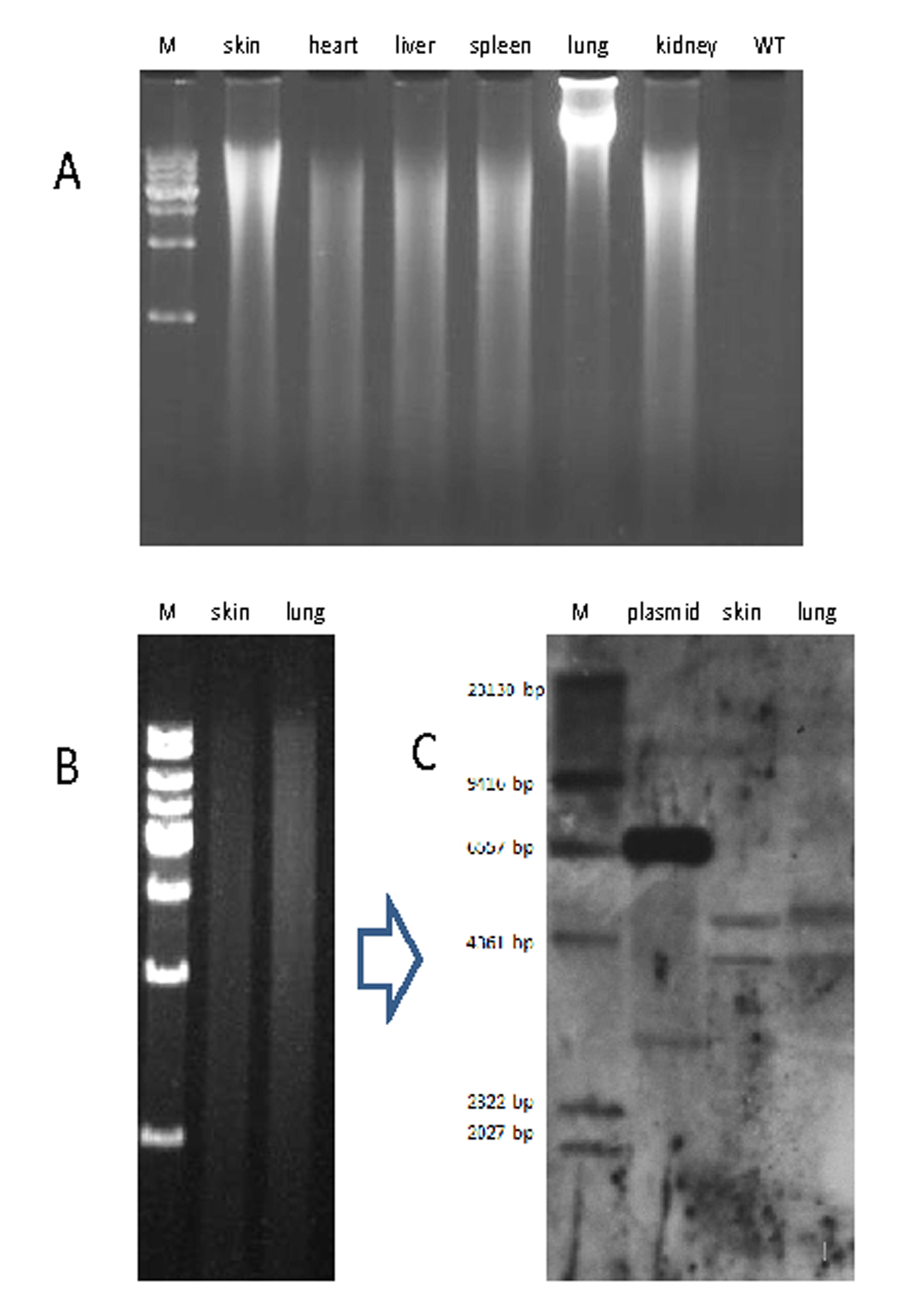

Supplement: Figure S6 — Repeated Southern blot analysis of transgene integration in the skin and lung of transgenic founder pig 1#. The genomic DNA samples of the skin and lung of founder pig1# were not thoroughly digested with PstI endonuclease in the first Southern blot assay (A). In the repeated Southern blot analysis, the same genomic DNA samples were completely digested as indicated by gel electrophoresis (B), and then transgene integration was detected by Southern blot in the two organs (C). M: DNA marker (1 Kb ladder in gel electrophoresis, and DNA molecular weight marker II in Southern blot assay). (TIF) [file pone.0108347.s006.tif]
